# Supplementary material for: Lack of Polysomnographic Non-REM Sleep Changes in Early Parkinson’s Disease
Source: Mov Disord. 2013 Jun 6;28(10):1443–6. doi: 10.1002/mds.25520 (PMC4238738; doi:10.1002/mds.25520)
Supplement: Supplementary file 1 [file mds0028-1443-SD1.pdf]

Lack of Polysomnographic Non-REM Sleep Changes in Early Parkinson's Disease

Nico J. Diederich, MD,[1,2,3]* Olivier Rufra, RN,[2] Vannina Pieri, MS,[1] Géraldine Hipp, MS,[1] and Michel Vaillant, PhD[4]

[1]*Department of Neuroscience, Centre Hospitalier de Luxembourg, Luxembourg-City, Luxembourg* [2]*Interdisciplinary Sleep Laboratory, Centre Hospitalier de Luxembourg, Luxembourg-City, Luxembourg* [3]*Centre for Systems Biomedicine, University of Luxembourg, Esch-Belval, Luxembourg* [4]*Methodology and Statistical Unit, CRP-Santé, Strassen, Luxembourg*

ABSTRACT

Background: Polysomnography (PSG) data are rare in patients who have early stage idiopathic Parkinson's disease (IPD).
Methods: Thirty-three patients who had IPD with a disease duration ≤3 years and 37 age-matched controls were recruited. PSG analysis was performed on current medication.
Results: Patients with IPD had a reduced mean percentage of muscle atonia during rapid eye movement (REM) sleep (80% vs 93%; $P < 0.05$). Total sleep time, sleep efficiency, indices/hour of arousals, awakenings, apnea/hypopnea, and periodic leg movements were similar in both groups. Age, but not dopaminergic medication, had a negative impact on sleep architecture in patients with IPD. There was no correlation between sleep efficiency assessed by PSG and sleep quality assessed by questionnaire.
Conclusions: The results confirmed a reduction in muscle atonia during REM sleep as a characteristic finding in early IPD. However, there were no further disease-inherent or medication-induced changes in sleep architecture. Although sleep disturbances are considered to be an integral part of IPD, PSG cannot yet identify them objectively at an early stage. © 2013 International Parkinson and Movement Disorder Society

Key Words: Parkinson's disease; polysomnography; REM sleep behavior disorder; sleep; sleep questionnaire

In idiopathic Parkinson's disease (IPD), disease-inherent degeneration of sleep regulation centers has been postulated, and rapid eye movement (REM) sleep behavior disorder has been identified as a forerunner syndrome.[1–4] With longer disease duration, polysomnography (PSG) has revealed progressive sleep "destructuring."[5] At an advanced stage of the disease, patients with IPD also have lower sleep efficiency and shorter REM stage than age-matched controls.[6] However, some of these findings have been collected in cohorts with variable disease duration and with preferential recruitment of the most disabled patients.[7] Thus, we lack objective PSG data on sleep quality and quantity in *early* motor stages of IPD.[7] The objective of the present study was to use PSG to search for REM sleep atonia in patients with early IPD, defined as a disease duration ≤3 years. Secondary objectives were to search for other objective sleep changes, to explore the impact of dopaminergic treatment on sleep quality and quantity, and, finally, to compare subjective sleep complaints with the PSG data.

Patients and Methods

Within a *prospective* study on early nonmotor signs in patients with IPD, we recruited nondemented IPD patients.[8] Disease duration <3 years was a strict inclusion criterion. Nondemented, healthy controls were recruited as nonconsanguineous family members or by the media. Controls underwent a brief medical checkup to exclude neurodegenerative or medical conditions, potentially endangering sleep quality. A 1-night PSG study was performed with a 16-channel montage, as previously described.[5,8] To avoid a medication withdrawal effect, patients and controls were permitted to stay on their usual medications. Levodopa (L-dopa) equivalent daily doses (LEDD) were calculated by using the following formula: 100 mg L-dopa = 1 mg of pergolide = 10 mg bromocriptine = 3 mg of ropinirole = 1 mg of pramipexole.[9] Sleep stage classification was performed by dividing sleep into stage R (REM sleep), stage N 1 (NREM 1) sleep, stage N 2 (NREM 2) sleep, and stage N 3 (NREM 3) sleep.[10] The following sleep

Additional Supporting Information may be found in the online version of this article.

*__Correspondence to:__ Dr. Nico Diederich, Centre Hospitalier de Luxembourg, 4, rue Barblé, L-1210-Luxembourg-City, Luxembourg; diederdn@pt.lu

Funding agencies: This study was supported by the Fonds National de Recherche (FNR/06/04/05), the Luxembourg Parkinson Association, and the Fondation Think, Luxembourg.

Relevant conflicts of interest/financial disclosures: Nothing to report. Full financial disclosures and author roles may be found in the online version of this article.

Received: 9 October 2012; **Revised:** 10 April 2013; **Accepted:** 22 April 2013

Published online 6 June 2013 in Wiley Online Library (wileyonlinelibrary.com). DOI: 10.1002/mds.25520

© 2013 The Authors. Movement Disorders published by Wiley on behalf of the International Parkinson and Movement Disorder Society. This is an open access article under the terms of the Creative Commons Attribution License, which permits use, distribution and reproduction in any medium, provided the original work is properly cited.

TABLE 1. Demographic and polysomnographic data of 33 IPD patients and 37 age-matched controls

Variable	Mean ± SD or No. (%)		
	IPD patients	Controls	P
Demographic data			
Age, y	65.5 ± 11.6	66.7 ± 9.0	0.66[a]
Sex: Men/women	12/21	21/16	0.09
Educational level, y	12.2 ± 3.7	12.6 ± 3.5	0.77[b]
MMSE score	28.82 ± 1.7	28.78 ± 3.0	0.95[b]
BMI, kg/m^2	25.3 ± 3.9	27.0 ± 4.0	0.08[b]
Hoehn-Yahr stage	2.0 ± 0.5	–	–
PD duration, y	1.9 ± 1.3	–	–
Levodopa dosage, mg	238.3 ± 331.5	–	–
Dopamine agonists, mg[c]	1.0 ± 2.0	0.06 ± 0.3	<0.01
Antidepressants	8 (24)	6 (16)	0.40
Sedatives	4 (12)	5 (13)	0.86
Total sleep time, min	308.8 ± 58.0	325.8 ± 63.5	0.24[b]
Polysomnographic data			
Total sleep time, min	308.8 ± 58.0	325.8 ± 63.5	0.24[b]
Sleep latency, min	36.7 ± 27.3	36.3 ± 26.5	0.95[a]
Sleep efficiency, %	72.1 ± 15.2	74.4 ± 12.9	0.49[a]
NREM sleep stage 2, min	175.2 ± 47.0	176.4 ± 51.8	0.98[d]
NREM sleep stage 2, %	43.4 ± 11.4	44.5 ± 13.3	0.61[b]
NREM sleep stage 3, min	51.2 ± 45.7	61.9 ± 47.14	0.34[a]
NREM sleep stage 3, %	13.6 ± 12.0	14.6 ± 9.2	0.69[a]
REM sleep stage, min	43.0 ± 29.6	53.7 ± 25.9	0.09[d]
REM sleep stage, %	11.1 ± 7.1	13.6 ± 7.1	0.15[a]
Index of apnea/hypopnea/h	2.5 ± 4.9	2.1 ± 3.3	0.71[a]
Index of periodic leg movements/h	4.7 ± 14.9	5.8 ± 13.2	0.75[a]
REM sleep muscle atonia, %	79.9 ± 30.9	92.7 ± 22.4	0.0497[a]
Awakenings/h	6.0 ± 4.0	4.5 ± 3.2	0.10[a]
Arousals/h	7.8 ± 4.8	7.9 ± 6.7	0.94[a]

[a]Welch analysis of variance.
[b]Mann-Whitney test.
[c]Expressed in pergolide equivalents, with 1 mg of pergolide = 10 mg bromocriptine = 3 mg of ropinirole = 1 mg of pramipexole.
[d]Analysis of variance (log values).
SD, standard deviation; IPD, idiopathic Parkinson's disease; MMSE, Mini-Mental State Examination; BMI, body mass index; PD, Parkinson's disease; NREM, non-rapid eye movement; REM, rapid eye movement.

parameters were evaluated: total sleep time; sleep latency; sleep efficiency; percentage of the different sleep stages; and indices per hour of sleep of apnea/hypopnea, periodic leg movements, awakenings, arousals. For these parameters, we used the definitions from another study published in this journal.[11] The percentage of muscle atonia during REM sleep was calculated according to Gagnon et al.[12] REM sleep was divided in epochs of 20 seconds. Loss of muscle atonia in an epoch was defined as the presence of ≥50% in that epoch of *any* chin electromyographic activity with an amplitude twice the amplitude measured during atonia and >10 μV.[12] All participants fulfilled the Parkinson's disease sleepiness scale (PDSS).[13] To estimate subjective sleep quality, we added the scores obtained on questions 1, 2, 4, 14, and 15 of the PDSS and defined that score as the subjective sleep quality score (SSQS). Statistical analysis used χ^2 or Fisher exact tests for categorical data. Analyses of variance (ANOVA) were used on raw values with a Welch adjustment in case of nonhomogeneity of variances between groups, or on log-transformed values for continuous outcomes, as appropriate. Nonparametric tests, such as the Mann-Whitney test, were applied when normality was not verified. In this exploratory study, the percentage of REM sleep atonia was designated as the primary outcome variable; all other outcome variables were secondary. Before entering the study, all patients provided informed written consent, and the study had been approved by the National Ethical Committee for Research.

Results

Demographic and Descriptive PSG Data in IPD Patients and Controls

Demographic data are presented in Table 1 and indicate that there was no significant difference between the patients with IPD and the control group. The PSG data are also shown in Table 1 and in Figure 1. The frequencies of different sleep dysfunction syndromes in both groups are presented in Supplemental Table 1. There was loss of muscle atonia during REM sleep in the patients with IPD ($P < 0.05$). It is noteworthy that there were no personal accounts of potential previous REM sleep behavior disorder, and no participant exhibited acting out of dreams during PSG. Patients with IPD had the same amount of total sleep, the same percentage of sleep efficiency, and similar distribution of the different sleep stages. When comparing only those patients with IPD who were not on antidepressants versus those controls who were not

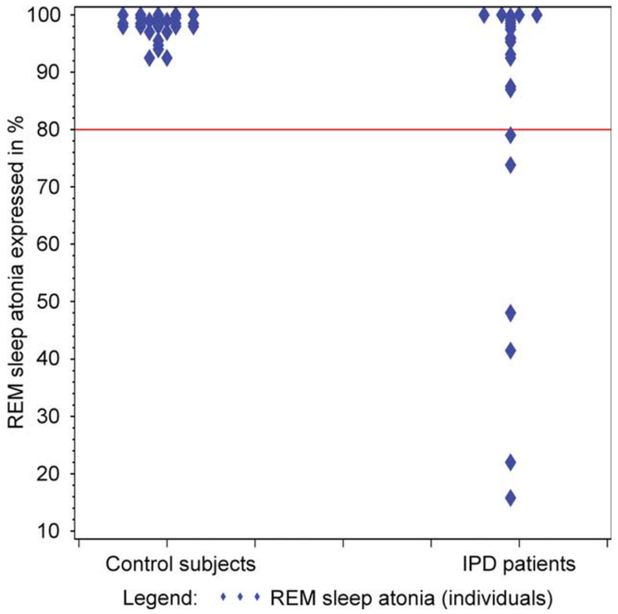

FIG. 1. Rapid eye movement (REM) sleep atonia is compared between 31 patients with idiopathic Parkinson's disease (IPD) and a group of 36 controls. Note that these numbers do not add up to the total numbers of patients and controls, because some individuals did not have any REM sleep).

on antidepressants, the absence of any difference was confirmed (data not shown). After omission of the nonsignificant interaction between age and diagnosis of IPD, there was an effect of age ($P = 0.001$; general linear model) on sleep efficiency. The negative correlation indicated decreasing sleep efficiency with increasing age, independent of IPD diagnosis (correlation coefficient $[r] = -0.67$; $P < 0.0001$). The apnea/hypopnea index was correlated with body mass index in both patients with IPD and controls.

The Effect of Age and Dopaminergic Treatment on Sleep Quality in Patients With IPD

In patients with IPD, age was linked to decreased percentages of total sleep time and REM sleep time and to an increased index of awakenings (see Supplemental Tables 2 and 3). L-dopa and dopamine agonists had a mild, but mostly statistically nonsignificant effect on total sleep time and sleep efficiency, with the former reducing these values and the latter increasing them (data not shown). However, when dopaminergic treatment was considered as a whole and expressed as the LEDD, no correlation was observed between the LEDD and the amount of NREM stage 2 or NREM stage 3 sleep ($r = -0.04$ [$P = 0.82$] and $r = -0.27$ [$P = 0.13$], respectively) (see Supplemental Table 4).

PDSS Questionnaire

Total scores on the PDSS were lower in the IPD patient group than in the control group (105.6 ± 22.0 vs 122.4 ± 15.4; $P = 0.0005$). In the patients with IPD, the SSQS score was not linked to sleep efficiency or with indices of arousals or awakenings ($r_{SSQS} = 0.08$, $r_{arousals} = -0.22$, and $r_{awakenings} = -0.18$).

Discussion

The current study had an exploratory character, because we recruited a limited number of patients, and a power analysis was not performed. Evidently, larger scale studies are warranted to confirm these results. However, the actual sample size needed is unknown, because "the effect size between various sleep disorders and IPD has not been established."[7] This restriction also applies to the effect size required when comparing IPD patients with healthy controls. Despite these limitations, our study produced several novel results, which may be counterintuitive at first glance. These results can be summarized by the statement that, surprisingly, at an early stage in IPD (defined as disease duration <3 years), objective sleep abnormalities still are very subtle. This statement is somehow in contradiction with Braak's model of an ascending degeneration in IPD. In this model, sleep-regulating centers, namely, the nucleus pedunculopontinus, locus coeruleus, and Raphé nuclei, are involved by the degenerative process ahead of the core motor syndrome. Thus, distinct clinical syndromes already should be present at an early stage. With this perspective, we could only confirm that some, but not all, patients with IPD have reduced percentages of muscle atonia during REM sleep.[3,12] However, the mean reduction in REM sleep atonia for our whole group of patients with IPD was a robust finding, This reduction was less marked than that reported by Gagnon et al., who studied patients with a longer disease duration. The applied scoring method produced reliable results, as illustrated by identical mean scores for muscle atonia in our controls and in those reported by Gagnon et al.[12] Alternatively, consideration of phasic chin or limb muscle activity has been strongly advocated,[14] although it remains controversial whether specificity can be increased by adding up different methods.[15] In the future, computer-assisted, automated quantification of loss of atonia, possibly in more than 1 muscle, may be the method of choice.[16,17] Negative ageing effects on sleep added up in patients with IPD more markedly than in age-matched controls, and a particularly significant interaction was observed between age and sleep efficiency.

PSG data on early stage PD are scarce, with only reports on small[18–20] and heterogeneous cohorts, because the results from patients with variable disease duration have often been presented together.[7] Patients with IPD in a middle disease stage have been compared with age-matched controls.[5,21–23] The mean disease duration in the largest comparable study[21] was 6.4 years, in contrast to 1.9 years in the current study. In concordance with our study, the authors of that study did not observe increased numbers of arousals, indices of apnea/hypopnea, or periodic leg movements in patients with IPD. However, in contrast to our study, the patients who had IPD with longer disease duration had shorter total sleep time and lower sleep efficiency than the controls. Thus, it is possible that, at the end of the motor "honeymoon period", usually estimated to be 5 years long sleep "destructuring" becomes visible.[5] Unfortunately, a remarkable PSG study in terms of recruitment (greater than 400 patients) did not produce comparable results, because it included IPD inpatients who had an extremely wide range of disease duration and without a comparison group.[24]

We based our findings on a 1-night PSG registration, and participants were not acclimatized to the environment of a sleep laboratory. However, this restriction equally applied to the patients with IPD and the controls. We cannot exclude the possibility that the medical treatment somehow tempered the impact of the neurodegenerative process on sleep architecture, although dopaminergic treatment considered as a whole was without any major impact on sleep quality or quantity. We also cannot exclude the possibility that the use of sedatives and antidepressants, in both

some patients and some controls, partially compensated for sleep fragmentation. Exclusive recruitment of de novo, drug-naive patients would have circumvented these potential elements of interference.

It has been puzzling to note that, in contrast to the rather reassuring PSG data, patients with early stage IPD had numerous subjective sleep complaints. A statistical link between the complaints and the objective sleep parameters could not be established. We are not aware of any other study that has compared subjective sleep complaints and PSG data at such an early stage in the disease. What are the possible causes for this discrepancy between subjective complaints and objective findings? It is possible that sleep questionnaires also register fatigue and exhaustion, which are not reflected by objective sleep data. It is also possible that in-depth PSG analysis or new evaluation tools, beyond routine PSG, would unmask subtle abnormalities that are undetectable using the current method. Promising methods have probed autonomic or sleep cycling regulation in sleep. Reduced heart rate variability as well as abnormalities in sleep microstructure have been reported.[18,25]

In conclusion, this study confirms reduction of muscle atonia during REM sleep as a characteristic finding in early IPD, but does not (yet) show evidence of any NREM sleep changes. Although sleep disturbances are considered to be an integral part of IPD, conventional PSG cannot yet objectify them at an early motor stage of the disease.

Acknowledgements: We thank Prof Dr. Mathias Maschke in Trier (Germany) and Drs. Stefan Beyenburg, Alexander Bisdorff, Pit Büchler, Dirk Droste, Marc Gleis, Bernd Wauschkuhn, all in Luxembourg, for the referral of patients for this study.

References

1. Arnulf I, Konofal E, Merino-Andreu M, et al. Parkinson's disease and sleepiness: an integral part of PD. Neurology 2002;58: 1019–1024.
2. Pal PK, Thennarasu K, Fleming J, Schulzer M, Brown T, Calne SM. Nocturnal sleep disturbances and daytime dysfunction in patients with Parkinson's disease and in their caregivers. Parkinsonism Relat Disord 2004;10:157–168.
3. Postuma RB, Lang AE, Massicotte-Marquez J, Montplaisir J. Potential early markers of Parkinson disease in idiopathic REM sleep behavior disorder. Neurology 2006;66:845–851.
4. Postuma RB, Gagnon JF, Rompre S, Montplaisir JY. Severity of REM atonia loss in idiopathic REM sleep behavior disorder predicts Parkinson disease. Neurology 2010;74:239–244.
5. Diederich NJ, Vaillant M, Mancuso G, Lyen P, Tiete J. Progressive sleep "destructuring" in Parkinson's disease. A polysomnographic study in 46 patients. Sleep Med 2005;6:313–318.
6. Shpirer I, Miniovitz A, Klein C, et al. Excessive daytime sleepiness in patients with Parkinson's disease: a polysomnography study. Mov Disord 2006;21:1432–1438.
7. Peeraully T, Yong MH, Chokroverty S, Tan EK. Sleep and Parkinson's disease: a review of case-control polysomnography studies. Mov Disord 2012;27:1729–1737.
8. Diederich NJ, Pieri V, Hipp G, Rufra O, Blyth S, Vaillant M. Discriminative power of different non motor signs in early Parkinson's disease. A case-control study. Mov Disord 2010;25: 882–887.
9. Hobson DE, Lang AE, Martin WR, Razmy A, Rivest J, Fleming J. Excessive daytime sleepiness and sudden-onset sleep in Parkinson disease: a survey by the Canadian Movement Disorders Group. JAMA 2002;287:455–463.
10. Silber MH, Anconi-Israel S, Bonnet MH, et al. The visual scoring of sleep in adults. J Clin Sleep Med 2007;3:121–131.
11. Diederich NJ, Vaillant M, Leischen M, Mancuso G, Golinval S, Nati R, Schlesser M. Sleep apnea syndrome in Parkinson's disease. A case-control study in 49 patients. Mov Disord 2005;20: 1413–1418.
12. Gagnon JF, Bedard MA, Fantini ML, et al. REM sleep behaviour disorder and REM sleep without atonia in Parkinson's disease. Neurology 2002;59:585–589.
13. Chaudhuri KR, Pal S, DiMarco A, et al. The Parkinson's disease sleep scale: a new instrument for assessing sleep and nocturnal disability in Parkinson's disease. J Neurol Neurosurg Psychiatry 2002; 73:629–635.
14. Frauscher B, Iranzo A, Gaig C, et al. Normative EMG values during REM sleep for the diagnosis of REM sleep behavior disorder. Sleep 2012;35:835–847.
15. Montplaisir J, Gagnon JF, Fantini ML, et al. Polysomnographic diagnosis of idiopathic REM sleep behavior disorder. Mov Disord 2010;25:2044–2051.
16. Frauscher B, Ehrmann L, Hogl B. Defining muscle activities for assessment of REM sleep behavior disorder: from a qualitative to a quantitative diagnostic level [published online ahead of print December 13, 2012]. Sleep Med 2012.
17. Fulda S, Plazzi G, Ferri R. Scoring atonia during normal and pathological rapid eye movement sleep: visual and automatic quantification methods [published online ahead of print February 26, 2013]. Sleep Biol Rhythms 2013.
18. Brunner H, Wetter TC, Hogl B, Yassouridis A, Trenkwalder C, Friess E. Microstructure of the non rapid eye movement sleep electroencephalogram in patients with newly diagnosed Parkinson's disease: effect of dopaminergic treatment. Mov Disord 2002;17: 928–933.
19. Buskova J, Klempir J, Majerova V, et al. Sleep disturbances in untreated Parkinson's disease. J Neurol 2011;258:2254–2259.
20. Garcia-Borreguero D, Caminero AB, De La Llave Y, Larrosa O, Barrio S, Granizo JJ, Pareja JA. Decreased phasic EMG activity during rapid eye movement sleep in treatment-naive Parkinson's disease: effects of treatment with levodopa and progression of illness. Mov Disord 2002;17:934–941.
21. Yong MH, Fook-Chong S, Pavanni R, Lim LL, Tan EK. Case control polysomnographic studies of sleep disorders in Parkinson's disease [serial online]. PLoS One 2011;6:e22511.
22. Cochen De Cock V, Abouda M, Leu S, et al. Is obstructive sleep apnea a problem in Parkinson's disease? Sleep Med 2010;11: 247–252.
23. Trotti LM, Bliwise DL. No increased risk of obstructive sleep apnea in Parkinson's disease. Mov Disord 2010;25:2246–2249.
24. Sixel-Doring F, Trautmann E, Mollenhauer B, Trenkwalder C. Associated factors for REM sleep behavior disorder in Parkinson disease. Neurology 2011;77;1048–1054.
25. Sauvageot N, Vaillant M, Diederich NJ. Reduced sympathetically driven heart rate variability during sleep in Parkinson's disease: a case-control polysomnography-based study. Mov Disord 2011;26: 234–240.
